# Case Report: A novel hemizygous nonsense variant in *PPP1R3F* gene associated with developmental delay, intellectual disability and autism spectrum disorder

**DOI:** 10.3389/fnins.2026.1998834

**Published:** 2026-10-30

**Authors:** Qi Yang, Qinle Zhang, Qiang Zhang, Xunzhao Zhou, Shang Yi, Zailong Qin, Sheng He, Jingsi Luo

**Affiliations:** 1 Guangxi Key Laboratory of Birth Defects Research and Prevention, Guangxi Key Laboratory of Reproductive Health and Birth Defects Prevention, Maternal and Child Health Hospital of Guangxi Zhuang Autonomous Region, Nanning, China; 2 Department of Genetic and Metabolic Central Laboratory, Maternal and Child Health Hospital of Guangxi Zhuang Autonomous Region, Nanning, China; 3 Guangxi Clinical Research Center for Birth Defects, Maternal and Child Health Hospital of Guangxi Zhuang Autonomous Region, Nanning, China; 4 Guangxi Clinical Research Center for Pediatric Diseases, Maternal and Child Health Hospital of Guangxi Zhuang Autonomous Region, Nanning, China

**Keywords:** neurodevelopmental disorder, novel variants, *PPP1R3F*, PPP1R3F deficiency syndrome, whole exome sequencing

## Abstract

**Background:**

PPP1R3F deficiency syndrome is an extremely rare, recently identified X-linked recessive neurodevelopmental disorder. It is caused by hemizygous variants in the *PPP1R3F* gene, which encodes a glycogen-targeting subunit of the brain-specific protein phosphatase 1 (PP1). Dysfunction of PPP1R3F disrupts glycogen metabolism in astrocytes, leading to insufficient energy supply to synapses during critical periods of brain development and subsequently causing neurodevelopmental abnormalities. Only a small number of cases of PPP1R3F deficiency syndrome have been reported to date, which significantly limits our comprehensive understanding of the clinical features of the disease and the relationship between genetic variants and clinical manifestations in patients.

**Methods:**

Whole-exome sequencing (WES) was performed on a Chinese male presenting with intellectual disability (ID), developmental delay (DD), and autism spectrum disorder (ASD). Sanger sequencing was then used to identify and determine the origins of the variant. We evaluated the pathogenicity of the identified variant following the established ACMG/AMP guidelines. Additionally, we conducted a thorough review of previously reported cases to summarize the variants and clinical features of PPP1R3F deficiency syndrome.

**Results:**

We identified a novel hemizygous nonsense variant [NM_033215.5: c.907C>T, p.(Gln303*)] in the *PPP1R3F* gene in the proband, which was inherited from his mother. The patient we described here exhibited clinical phenotypes similar to those associated with *PPP1R3F* gene variants but lacked other reported multisystem manifestations, such as epilepsy, brain abnormalities, and dysmorphic features. An analysis of the 15 variants and their associated phenotypes in 15 patients from both the current and previous studies demonstrates that *PPP1R3F* gene variants can lead to a variety of phenotypic deficits, and a clear genotype–phenotype correlation has not yet been established. Despite the high variability in some clinical manifestations, the core phenotypic features of *PPP1R3F* variants include varying degrees of ID, DD, speech delay, dysmorphic facial features, and ASD.

**Conclusion:**

Our findings support DD, ID, ASD, and speech impairment as core features of PPP1R3F deficiency syndrome, while also highlighting the extensive clinical variability. This study further expanded the genetic and phenotypic spectrum associated with PPP1R3F deficiency syndrome.

## Introduction

PPP1R3F deficiency syndrome is a rare X-linked recessive neurodevelopmental disorder (Liu et al., 2023; Piton et al., 2011). It is characterized by DD, mild to severe ID, ASD, epilepsy, hypotonia, gait instability, and cerebellar ataxia (Liu et al., 2023). The disorder is caused by hemizygous variants in the protein phosphatase 1 regulatory subunit 3F gene (*PPP1R3F*, NM_033215.5, MIM 301104), which is located at Xp11.23 region and encodes the glycogen-targeting subunit of brain-specific protein phosphatase 1 (PP1). As a master regulator of cellular activity, PP1 requires association with glycogen-targeting subunits (GTSs) to orchestrate glycogen metabolism (Cohen, 2002; Hendrickx et al., 2009; Verbinnen et al., 2017; Costello et al., 2017). PPP1R3F, the most brain-specific member of the GTS family, is constitutively anchored to the astrocytic endoplasmic reticulum membrane through its C-terminal transmembrane domain, serving as a “gatekeeper” for cerebral glycogen homeostasis (Kelsall et al., 2009, 2011; Alsina et al., 2019; Costello et al., 2017). Loss-of-function variants in *PPP1R3F* disrupt its interaction with PP1, reducing protein stability and causing intracellular mislocalisation. This molecular defect impairs the ability of PPP1R3F to direct PP1 in coordinating glycogen synthesis and breakdown, thereby preventing astrocytes from accumulating glycogen during glucose surplus and mobilising reserves during metabolic stress. The consequent failure of glycogen-dependent energy buffering disrupts the astrocytic glycogen-lactate axis, upon which developing neurons critically rely for ATP generation and synaptic plasticity signalling. This energy deficit directly compromises synaptic transmission and neural circuit maturation, ultimately translating the underlying metabolic disturbance into the clinical phenotype of neurodevelopmental disorder (Piton et al., 2011).

To date, only two international studies have systematically reported PPP1R3F deficiency syndrome, which together included 14 male patients from 14 unrelated families across different regions worldwide through the GeneMatcher platform (Liu et al., 2023; Piton et al., 2011; Sobreira et al., 2015). These patients exhibited multisystem abnormalities, including developmental delay (DD), intellectual disability (ID), delayed language development, behavioral abnormalities, dysmorphic facial features, seizures, brain abnormalities, microcephaly, and skeletal and limb abnormalities. However, the number of cases worldwide remains extremely small, and clinical data across different ethnic groups and variant types are limited, which restricts our understanding of the phenotypic spectrum and the effects of these variants. Therefore, further reporting of *PPP1R3F* variants and their associated phenotypes is crucial for clarifying the natural history of the disease, establishing genotype–phenotype associations, and developing intervention strategies. Herein, we identified a novel nonsense variant in the *PPP1R3F* gene in a Chinese family with neurodevelopmental disorders. We also delineated the clinical characteristics of this case in detail and reviewed the phenotypic spectrum associated with PPP1R3F deficiency syndrome. This study expands the mutational profile of the *PPP1R3F* gene, contributing to a deeper understanding of PPP1R3F deficiency syndrome.

## Materials and methods

### Patients

A Chinese patient from a family with autism and developmental delay was referred to the Paediatric Rehabilitation Department of Guangxi Maternal and Child Health Hospital for genetic evaluation (Figure 1). The study protocol was approved by the Institutional Review Board and Ethics Committee of the Guangxi Maternal and Child Health Hospital and the study conducted in accordance with the principles of the Declaration of Helsinki. Written informed consent was obtained from the parents of the affected individuals for the publication of clinical data and patient photographs.

**Figure 1:**
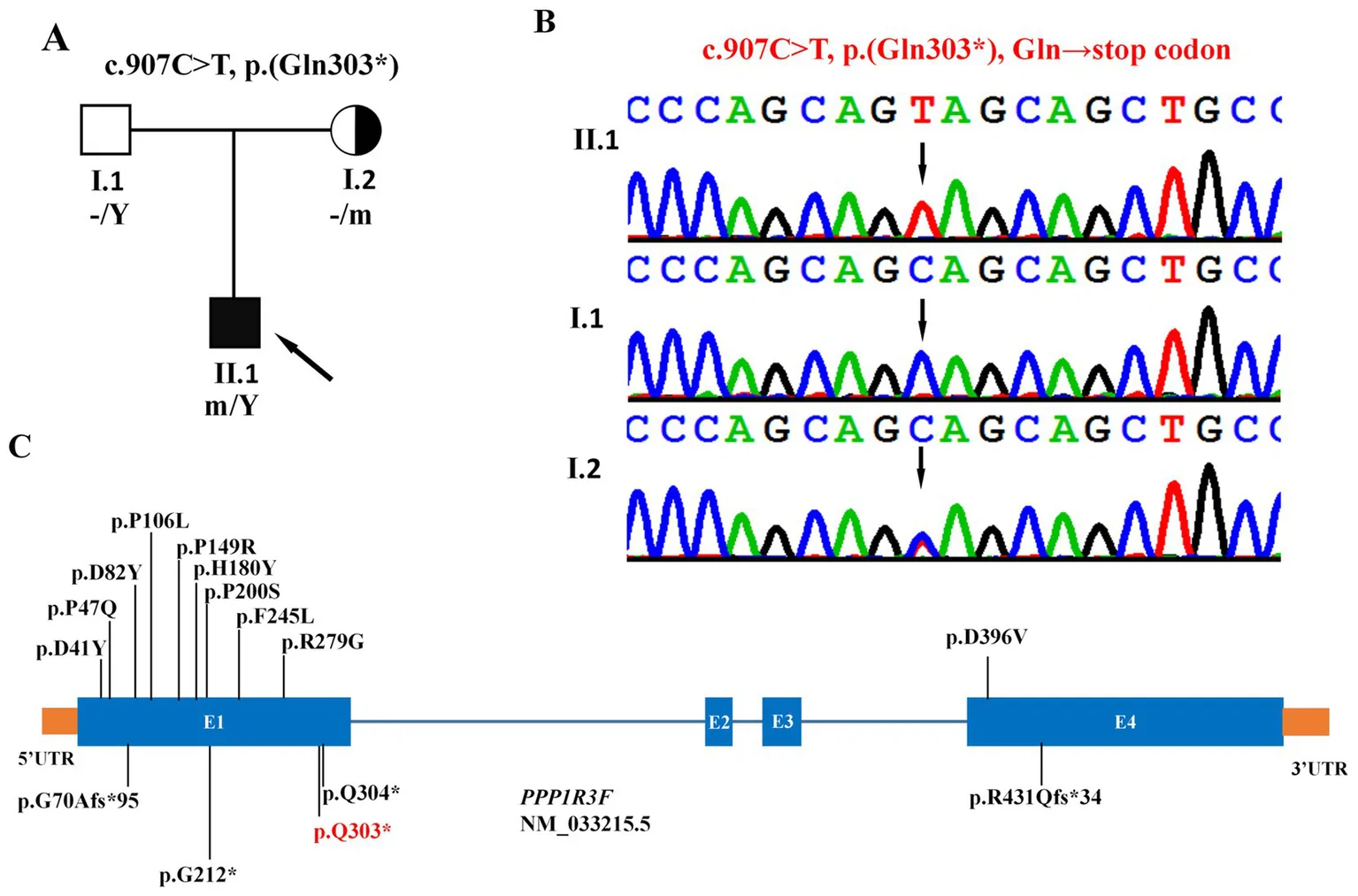
**(A)** Pedigree of the affected family. The proband was denoted by arrows. m, mutant allele; −, normal allele; Y, Y chromosome. **(B)** DNA sequence chromatograms from Sanger sequencing of *PPP1R3F*, showing a novel hemizygous variant c.907C>T, p.(Gln303*) in the proband (II-1). Further Sanger sequencing revealed that his mother was a heterozygous carrier (I:1), while his father did not carry the variant (I:2). **(C)** The distribution of all *PPP1R3F* variants detected so far in the 15 reported patients. The red variant represent the novel variants identified in this study. Boxes represent the four different exons, and solid lines connecting these boxes represent the introns of *PPP1R3F* gene.

### Whole exome sequencing and sanger sequencing

A Chinese family with autism and developmental delay was referred for genetic evaluation (Figure 1). Five millilitres of peripheral blood lymphocytes were collected from the patient and his family members. Genomic DNA was extracted from these samples using a Lab-Aid DNA kit (Zeshan Biotechnology Co., Ltd., Xiamen, China), following the manufacturer’s protocol. Whole-exome sequencing (WES) was performed using an Agilent SureSelect V5 enrichment capture kit (Agilent Technologies, Santa Clara, California, USA), followed by 100 bp paired-end sequencing on an Illumina HiSeq 2000 platform (Illumina, San Diego, California, USA). Sequencing reads were aligned to the human reference genome (GRCh38) using BWA-MEM v0.7.17, with default parameters. Duplicate reads were marked, and base quality score recalibration, variant calling, and joint genotyping were performed using GATK (version 3.4; Broad Institute, Cambridge, MA, USA). Variant annotation was performed using TGex software, prioritising variants with a minor allele frequency (MAF) of ≤0.001 in public databases such as the 1,000 Genomes Project, the NHLBI Exome Sequencing Project, and the Exome Aggregation Consortium. The functional effects of the candidate variants were then evaluated using in silico tools (REVEL, PolyPhen-2, SIFT, LRT, Mutation Assessor, CADD and MutationTaster). The Swiss-Model server^1^ was used to predict the three-dimensional (3D) structures of the wild-type and mutant PPP1R3F proteins. Finally, variant pathogenicity was interpreted according to the guidelines of the American College of Medical Genetics and Genomics (ACMG) and the ClinGen Sequence Variant Interpretation (SVI) Working Group (Richards et al., 2015).

## Result

The proband (II:1) is a boy of 4 years and 6 months with healthy, unrelated Chinese parents (Figure 1A). He was referred to the Paediatric Rehabilitation Department of Guangxi Maternal and Child Health Hospital for autistic behavior and language delay. He was born full term at 39 + 4 weeks gestation via a normal vaginal delivery. His birth weight was normal (3.24 kg). He had a global delay in the achievement of developmental milestones, including key milestones in motor skills, speech and language, and cognitive skills. He started walking at 20 months and had hypotonia. Language development was severely delayed. At 24 months, the child could speak only a few words, at 3 years, he could speak short sentences but still not long sentences. At the age of 3 years and 5 months, the Gesell Developmental Diagnostic Scale was used to assess his Developmental Quotient (DQ, DQ < 70 as low score) (gross motor 50.4, fine motor 50.4, adaptive 37.2, language 17, personal-social 33.2). He also exhibited autistic behaviors, including cognitive impairment, poor eye contact, repetitive behaviors, social anxiety, unusual fixations and obsessions, impulsivity, violent, and autistic-like behaviors. At the chronological age of 3 years and 4 months, standardized assessments demonstrated the following results: Kanner’s Behavioral Scale (KBS) score 20 (cutoff ≥14 for autism suspicion), Childhood Autism Rating Scale (CARS) score 37 (range 30–60, indicative of severe autism spectrum disorder), and Autism Behavior Checklist (ABC) score 71 (clinically significant threshold ≥67; DSM-5 code 299.00). Ultimately, the patient was diagnosed with autism spectrum disorder by a qualified clinician based on DSM-5 criteria (299.00). On his most recent examination at the age of 4 years and 6 months, he was found to have moderate intellectual disability [Wechsler Preschool and Primary Scale of Intelligence-IV (WPPSI-IV), Full Scale IQ = 41]. A physical examination revealed that he had mild short stature (96 cm, <−2SD), and microcephaly (head circumference of 48 cm, <−2 SD), and a low frontal hairline. Electroencephalogram (EEG) and magnetic resonance image (MRI) of the brain were normal and no abnormalities were found.

### Mutation analysis

Whole-exome sequencing (WES) was performed on the proband to identify the pathogenic variant underlying the condition. The run generated approximately 7 Gb of sequence reads, with a mean coverage of 98.8% of the target region and 97.6% of bases covered at a depth of at least 20×. A total of 25,556 coding or splice-site variants (within ±10 bp), including single nucleotide variants (SNVs) and insertions/deletions (indels), were identified. Filtering for rare variants (minor allele frequency (MAF) ≤ 0.001 in gnomAD, dbSNP132, the Exome Sequencing Project (ESP), the 1,000 Genomes Project, and an in-house database) reduced this number to 731 unique SNVs. After removing synonymous and in silico-predicted benign missense changes, 531 candidate variants remained (Supplementary Figure S1). Phenotype-driven analysis with TGex^2^ highlighted four candidate genes: *PPP1R3F*, *ALDOA*, *KANK1*, and *ZNF142*. Heterozygous variants in *ALDOA*, *KANK1*, and *ZNF142* were excluded because the associated disorders follow autosomal recessive inheritance. Finally, a hemizygous *PPP1R3F* nonsense variant [c.907C>T, p.(Gln303*)] was identified in the patient, and Sanger sequencing confirmed that his mother carried the variant, while no other family members did (Figure 1B). Differences in protein 3D structures were observed between the wild-type and mutant forms (Figure 2).

**Figure 2:**
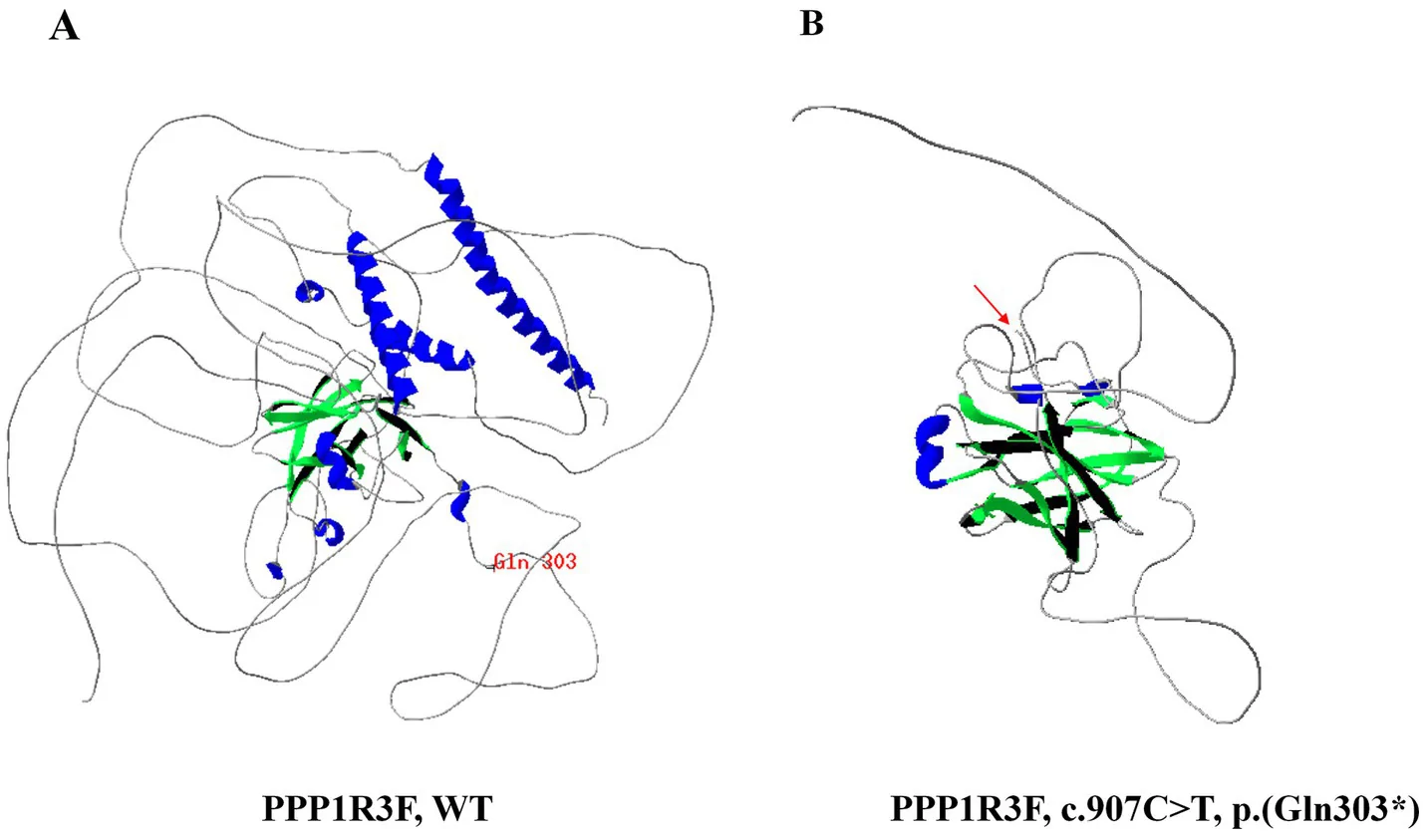
Analyses of *PPP1R3F* protein three-dimensional structure. **(A)** Wild-type, **(B)** c.907C>T, p.(Gln303*) mutant-type. The arrows indicate the location of p.(Gln303*), which shows a loss of the C-terminal transmembrane region downstream.

## Discussion

PPP1R3F (protein phosphatase 1 regulatory subunit 3F) is a glycogen-targeting subunit encoded by the *PPP1R3F* gene located on the X chromosome; it is part of the protein phosphatase 1 (PP1) holoenzyme complex (Liu et al., 2023). It regulates the substrate specificity and subcellular localization of the full-length enzyme complex (Cohen, 2002). Variants in the *PPP1R3F* gene were initially identified by whole-exome sequencing (WES) in a large autism cohort lacking detailed clinical descriptions (Piton et al., 2011). Subsequently, Liu et al. identified hemizygous variants in the *PPP1R3F* gene in 13 unrelated males and associated them with a novel X-linked recessive neurodevelopmental disorder. This disorder is characterised by DD, ID, ASD, seizures and other neurological abnormalities (Piton et al., 2011). In the present study, A hemizygous nonsense variant [c.907C>T, p.(Gln303*)] was identified in the coding region of *PPP1R3F* in a Chinese patient with a neurodevelopmental disorder. Further Sanger sequencing revealed that his mother was a heterozygous carrier, whereas his father did not carry the variant. To the best of our knowledge, this variant has not been reported previously and is not present in public databases, such as the NHLBI Exome Sequencing Project, gnomAD, 1,000 Genomes Project, Single Nucleotide Polymorphism Database, and disease-related databases like ClinVar and the Human Gene Mutation Database. MutationTaster predicts that the novel nonsense variant c.907C>T, p.(Gln303*) is disease-causing. Previous studies have shown that loss-of-function (LoF) variants in the *PPP1R3F* gene cause an X-linked neurodevelopmental disorder known as PPP1R3F deficiency syndrome. Truncating variants in this gene, including p.(Gln304*), markedly reduce protein abundance and eliminate the C-terminal transmembrane domain, which is responsible for anchoring PPP1R3F to the endoplasmic reticulum (ER) membrane (Liu et al., 2023). The c.907C>T p.(Gln303*) variant is located in exon 1 of the *PPP1R3F* gene. Protein structure modeling indicates that this variant results in the loss of the C-terminal transmembrane domain in the mutant protein. Similar to the loss-of-function variants reported previously [e.g. p.(Gln304*)], the p.(Gln303*) variant is predicted to trigger nonsense-mediated mRNA decay, leading to haploinsufficiency or to produce a severely truncated protein lacking the functional C-terminal transmembrane anchor (Figure 2) (Liu et al., 2023). This would disrupt the precise localization of the PPP1R3F-PP1 holoenzyme to the astrocytic endoplasmic reticulum membrane, a prerequisite for its role as a metabolic gatekeeper (Liu et al., 2023). Consequently, the variant is expected to impair the regulation of glycogen synthase and glycogen phosphorylase, leading to dysfunctional glycogen metabolism and disrupted astrocyte-neuron lactate shuttling (Kelsall et al., 2011). The c.907C>T, p.(Gln303*) variant was classified as likely pathogenic based on PVS1 criterion (null variant in a gene with established loss-of-function mechanism) and PM2_Supporting (absent from population databases), in accordance with the ACMG/AMP guidelines for interpretation of sequence variants (Table 1).

**Table 1:** Predicted pathogenicity of novel *PPP1R3F* variant.

Gene	Variant (NM_005121.3)	Inheritance	AutoPVS1	Mutationtaster	RDDC	ENTPRISE	ACMG/AMP
PPP1R3F	c.907C>T, p.(Gln303*)	Maternal	Very Strong	D	LP	D	LP (PVS1 + PM2_Supporting)

D, deleterious or damaging; LP, Likely pathogenic.

A summary of the reported *PPP1R3F* variants and the associated clinical features across all documented cases is presented in Table 2. Phenotypic analysis of affected individuals revealed considerable heterogeneity among patients carrying *PPP1R3F* variants. Nevertheless, certain features were observed in more than half of the cases. All 15 patients exhibited ID and/or DD, though the severity was generally mild, with only three individuals showing moderate to severe impairment. In terms of motor function, all patients learn to walk, but they also experience hypotonia, hyperreflexia, spasticity in the lower limbs, dystonia and ataxia. Our patient also exhibited moderate ID and delayed motor development. Language development appears to be the most vulnerable domain, as all patients showed delayed language acquisition. Over a third of patients had no language skills, and most also displayed notable impairments in expressive language. Behavioral abnormalities were observed in 10 out of 12 patients, including autism spectrum disorder (ASD), attention deficit hyperactivity disorder (ADHD), and aggressive behavior. Facial dysmorphism was present in approximately 83.3% of cases (10 out of 12), and these characteristics appeared to become increasingly prominent with age; however, no common facial pattern was observed. The manifestations ranged from subtle uplifted earlobes to extensive facial malformations. No facial deformities were observed in the remaining two patients (including our patient). Seizures occurred in 7 out of 15 patients and manifested in various forms, including generalized, nocturnal, tonic, atonic, focal myoclonic, and atypical absence seizures. Most patients responded well to pharmacological intervention, with effective seizure control achieved through either monotherapy (e.g., valproate) or combination treatments. Interestingly, some patients who never had overt seizures still demonstrated interictal electroencephalographic (EEG) abnormalities, suggesting a potential risk for future epileptogenesis. This underscores the need for longitudinal monitoring to assess whether such subclinical EEG changes predict later seizure onset. Importantly, although seizures and intellectual disability (ID) often co-occur, their relationship is complex. Some patients with ID do not develop seizures, and even among those whose seizures are well-controlled, cognitive function does not show corresponding improvement. Skeletal and limb abnormalities were observed in 5 of 13 patients, including joint hypermobility, ligamentous laxity, limb pain, pes planus, thoracolumbar scoliosis, mild clinodactyly of the fourth and fifth fingers, progressive flexion contracture of the fingers, camptodactyly, stiff feet, kyphosis, scoliosis, and pectus excavatum. Microcephaly and short stature were each seen in five out of fourteen patients. Brain MRI abnormalities were detected in four patients, although the nature of these anomalies varied. Other heterogeneous malformation features included occasional tremors, nocturnal enuresis, bruising susceptibility, dental crowding, dry skin, hypercalciuria, chronic fatigue, exercise intolerance, oculomotor apraxia, sleep disorder, small subependymal haemorrhage on the right side, deafness, myopia, female fat distribution, ventricular septal defect, valvular pulmonary stenosis, prematurity, growth retardation, conductive hearing loss, nystagmus, unsteady upward gaze, rigidity, being underweight, and a right undescended testis. In contrast, the patient described in this study did not exhibit these diverse dysmorphic features; specifically, no facial dysmorphisms, skeletal or limb deformities, seizures, brain MRI abnormalities, or other rare congenital anomalies were observed.

**Table 2:** Summary of reported information on clinical phenotypes in association with variants in patients with PPP1R3F deficiency syndrome.

Patients clinical data	Our patient	Piton et al. (2011)	Liu et al. (2023)													Total
Patients	1	2	3	4	5	6	7	8	9	10	11	12	13	14	15	*N* = 15
Age	4Y6M	NA	15Y	2.5Y	14Y	5Y	3Y	15 M	15Y	33Y	13Y	5Y	5Y	6Y	9Y	
Gender	Male	Male	Male	Male	Male	Male	Male	Male	Male	Male	Male	Male	Male	Male	Male	
PPP1R3F (NM_033215.5) variants	c.907C>T, p.(Gln303*)	c.733T>C, p.(Phe245Leu)	c.140C>A, p.(Pro47Gln)	c.446C>G, (p.Pro149Arg)	c.634G>T,(p.Gly212*)	c.910C>T, p.(Gln304*)	c.121G>T, p.(Asp41Tyr)	c.835C>G,p.(Arg279Gly)	c.538C>T,p.(His180Tyr)	c.317C>T, p.(Pro106Leu)	c.1290dupC,p.(Arg431Glnfs*34)	c.244G>T, p.(Asp82Tyr)	c.1187A>T, p.(Asp396Val)	c.207_228del, p.(Gly70Alafs*95)	c.598C>T, p.(Pro200Ser)	Nonsense = 3, Framshift = 2, Missense = 10
Learning disability	+	+	slow processing	+	+	+	+	+	+	+	+	Moderate	+	+	+	15/15
ID	Moderate	NA	Mild	Mild	Mild	−	NA	Mild	+	Severe	Severe	NA	Moderate	Mild	Mild	11/12
Speech delay	+	NA	+	+	+	+	Nonverbal	Nonverbal	NA	NA	Nonverbal	Nonverbal	Nonverbal	+	+	12/12
Behavioral disorder	ASD	ASD	Aggressive behavior	NA	ADHD	−	ASD	NA	Aggressive behavior	Aggression fits, bites hands when tense, otherwise social and friendly	Hand flapping	ASD	NA	ASD	−	10/12
Neurological manifestations	Mild motor delay	NA	Unsteady gait with increased deep tendon reflexes of lower extremities	Mild hyperkinesia	Gross motor DD, mixed hyper-hypotonia with brisk reflexes and abnormal gait	Gross motor DD; history of hypotonia and reduced core strength	Oral motor weakness	Spastic-dystonic quadriplegic cerebral palsy	−	pyramidal signs	Spastic paraplegia	Mild/moderate motor delay	Hypertonia mainly in lower limbs and brisk reflexes	Muscular hypotonia, fatigability, uses wheelchairs for longer distances	−	12/14
Dysmorphic Features	−	NA	−	+	+	+	+	NA	−	+	+	+	+	+	+	10/12
Seizures	−	+	Generalized	−	−	−	−	−	Generalized tonic, atonic, myoclonic and absence	−	Complex focal seizures	Atypical febrile seizures	Focal-myoclonic; Generalized	Myoclonic seizures, atypical absences, initially frequent and fever associated	−	7/15
Abnormal EEG	−	NA	NA	−	+	−	−	−	+	−	NA	−	−	NA	−	2/10
Brain MRI Abnormalities	−	NA	−	−	−	−	−	−	+	NA	+	+	+	−	−	4/13
Microcephaly	+	NA	−	−	+	−	−	+	−	−	+	+	−	−	−	5/14
Skeletal and limb abnormalities	−	NA	−	−	+	+	+	NA	−	+	−	−	−	+	−	5/13
Short stature	+	NA	−	−	+	−	−	−	−	+	+	+	−	−	−	5/14
Other			Occasional tremors, nocturnal enuresis		Bruising susceptibility, dental crowding, dry skin, hypercalciuria,	Chronic fatigue, exercise intolerance, oculomotor apraxia.	Sleep disorder,small subependymal hemorrhage on the right side			Deafness, myopia, female fat distribution	Ventricular septal defect, valvular pulmonary stenosis	Prematurity, growth retardation, conductive hearing loss	Nystagmus, occasional tremors, unsteady, upward gaze, rigidity, underweight, right undescended testis.	Sleep disturbance		
Other genetic findings					SMARCC2, c.230C>T (p.Pro77Leu),	SMARCC2, c.230C>T (p.Pro77Leu)										

Y, years; M, months; ID, intellectual disability; NA, not available; ASD, autism spectrum disorder; ADHD, attention deficit hyperactivity disorder; MRI, magnetic resonance image; EEG, electroencephalogram.

To date, variants in the *PPP1R3F* gene have only been documented in fifteen affected individuals from fifteen distinct families (Liu et al., 2023). These variants include two frameshift variants, ten missense variants, and two nonsense variants (Figure 1C). Notably, over 86.7% (13 out of 15) of the variants are located within exon 1, suggesting that this region acts as a mutational hotspot. The type of genetic variant often correlates with the severity of the associated clinical phenotype. A comparison of patient phenotypes revealed that, although patients with missense variants tended to exhibit more severe features, such as profound intellectual disability (ID), delayed language acquisition, increased seizure rates, brain structural abnormalities, and dysmorphic features, this trend did not reach statistical significance. Furthermore, the frequencies of other variable phenotypic features, such as behavioral abnormalities, short stature, and skeletal and limb malformations, did not show a significant association with the type of variant. Importantly, even patients carrying identical variants can display markedly different clinical presentations. For instance, the same type of missense variant in unrelated families may manifest as mild-to-severe developmental delay accompanied by ASD, or it may be accompanied by severe ID, epilepsy, brain abnormalities, and skeletal malformations (Liu et al., 2023). Therefore, individuals with hemizygous *PPP1R3F* variants demonstrate considerable clinical heterogeneity, and no definitive genotype–phenotype correlation has yet been established.

The mechanisms by which these variants lead to these variable clinical features have yet to be elucidated. These underlying mechanisms involve both disturbances to glycogen metabolism and non-metabolic regulatory abnormalities. The *PPP1R3F* gene encodes the glycogen-targeting subunit R3F, which is highly expressed in astrocytes and specifically anchored to the endoplasmic reticulum (ER) membrane in a tail-anchored manner. Through its canonical N-terminal RVxF motif, R3F recruits protein phosphatase 1 (PP1), leading to the dephosphorylation and subsequent activation of glycogen synthase (GS), thereby linking glycogen metabolism to neuronal glucose supply (Liu et al., 2023; Kelsall et al., 2011). Functional studies have shown that missense variants located within the PP1-binding domain [e.g., p.(Asp41Tyr) and p.(Pro47Gln)] disrupt PP1 binding by either breaking salt bridges or altering domain conformation. This results in the hyperphosphorylation of GS at GSK-3 phosphorylation sites (Ser640/644), which ultimately inhibits glycogen synthesis (Liu et al., 2023; Kelsall et al., 2011). However, not all missense variants cause glycogen metabolic defects. For instance, functional assays of p.(Pro106Leu), p.(His180Tyr), and p.(Arg279Gly) revealed no glycogen metabolic abnormalities, strongly suggesting that these variants may exert pathogenic effects through interactions with other, as-yet-unidentified substrates or regulatory proteins (Liu et al., 2023). Furthermore, truncating variants (e.g., p. Gln304Ter and p. Gly212Ter) lead to markedly reduced protein levels and loss of endoplasmic reticulum (ER)-anchoring capacity due to deletion of the C-terminal transmembrane domain. This impairs effective binding to PP1 and GS, and also results in the abnormal localisation of proteins to the nucleus, potentially due to the exposure of a cryptic nuclear localisation signal upon C-terminal truncation (Liu et al., 2023). The loss of ER localisation prevents the R3F–PP1 complex from residing in its normal subcellular environment, thereby disrupting interactions with ER-associated regulatory proteins and signalling pathways. Unlike other soluble glycogen-targeting subunits (e.g., R3C, R3D and R3G), R3F is unique in that it binds to the ER membrane via a C-terminal tail-anchored domain. This unique localisation enables R3F to direct PP1 enzymatic activity to the ER surface, rendering it susceptible to regulation by ER-associated kinases and calcium signalling (Liu et al., 2023). Moreover, transcriptional network analyses have identified PPP1R3F as a master regulator candidate for autism spectrum disorder (ASD), capable of modulating numerous downstream genes and endocytic pathways (Piton et al., 2011; Doostparast Torshizi et al., 2018). As other members of the GTS family (e.g., R3A) have been reported to dephosphorylate non-glycogen substrates, including the ryanodine receptor 2 (RyR2), it is possible that R3F functions in a similar way by regulating various ER-localised phosphorylated substrates such as astrocyte-specific RyRs or other neurodevelopment-related proteins (Liu et al., 2023). Taken together, *PPP1R3F* variants may contribute to a range of neurological phenotypes, including developmental delay, intellectual disability, epilepsy, and autistic features, through dual mechanisms involving ‘energy crisis’ and ‘oxidative stress’, as well as non-metabolic transcriptional dysregulation. Further case reports, genotype–phenotype correlation studies and functional analyses targeting specific variants are essential to fully elucidate the molecular basis of this disorder.

This study is subject to several limitations. Firstly, given the small number of reported patients, the genotype–phenotype observations in this study should be considered exploratory. Expanding the patient cohort would help to further elucidate the genotype–phenotype associations. Secondly, although previous functional studies have demonstrated that specific *PPP1R3F* missense variants interfere with PP1 binding and glycogen metabolism, and that other mutations may act through non-metabolic mechanisms, no functional experiments were performed in the present study. Consequently, it remains unresolved whether the p.(Gln303*) variant leads to nonsense-mediated mRNA decay (NMD), yields a truncated protein, or impairs endoplasmic reticulum anchoring and glycogen metabolism. Accordingly, the proposed mechanistic model involving astrocytic glycogen dysfunction, synaptic energy deficiency, and oxidative stress should be considered hypothetical and requires validation through future experimentation.

In summary, we identified a novel hemizygous likely pathogenic variant in the *PPP1R3* gene in a Chinese patient diagnosed with PPP1R3F-related neurodevelopmental disorder. The variant is predicted to undergo nonsense-mediated decay and cause loss of function, thereby expanding the mutational spectrum of the disease. The patient presented with core features of PPP1R3F deficiency syndrome, including DD, ID, autistic behaviors, and delayed speech. Importantly, unlike previously documented cases, this individual did not present with dysmorphic features, seizures, brain abnormalities, or skeletal malformations. Our study enhances current clinical understanding by synthesizing and analyzing phenotypic information from all 15 patients reported thus far. While neurodevelopmental impairment, behavioral issues, and language delay appear consistently present in PPP1R3F deficiency syndrome, the overall phenotype demonstrates significant heterogeneity, and no definitive genotype–phenotype relationship has yet been established. This report further highlights the importance of documenting variants and detailed clinical presentations from diverse populations in order to refine the clinical spectrum of the disease, understand its pathophysiology and ultimately guide the diagnosis and management of this rare neurodevelopmental disorder.

## Data Availability

The datasets presented in this study are available for consultation in online repositories. The names of the repository/repositories and accession number(s) can be found at the following URL: https://www.ncbi.nlm.nih.gov/sra/PRJNA1544735.

## Footnotes

1
https://swissmodel.expasy.org/

2
https://tgex.genecards.cn/

## References

[ref1] Alsina K. M. Hulsurkar M. Brandenburg S. Kownatzki-Danger D. Lenz C. Urlaub H. . (2019). Loss of protein phosphatase 1 regulatory subunit PPP1R3A promotes atrial fibrillation. Circulation 140, 681–693. doi: 10.1161/CIRCULATIONAHA.119.039642, 31185731 PMC6699925

[ref2] Cohen P. T. (2002). Protein phosphatase 1--targeted in many directions. J. Cell Sci. 115, 241–256. doi: 10.1242/jcs.115.2.241, 11839776

[ref3] Costello J. L. Castro I. G. Camões F. Schrader T. A. McNeall D. Yang J. . (2017). Predicting the targeting of tail-anchored proteins to subcellular compartments in mammalian cells. J. Cell Sci. 130, 1675–1687. doi: 10.1242/jcs.200204, 28325759 PMC5450235

[ref4] Doostparast Torshizi A. Duan J. Wang K. (2018). Transcriptional network analysis on brains reveals a potential regulatory role of PPP1R3F in autism spectrum disorders. BMC. Res. Notes 11:489. doi: 10.1186/s13104-018-3594-0, 30016992 PMC6050725

[ref5] Hendrickx A. Beullens M. Ceulemans H. Den Abt T. Van Eynde A. Nicolaescu E. . (2009). Docking motif-guided mapping of the interactome of protein phosphatase-1. Chem. Biol. 16, 365–371. doi: 10.1016/j.chembiol.2009.02.012, 19389623

[ref6] Kelsall I. R. Rosenzweig D. Cohen P. T. (2009). Disruption of the allosteric phosphorylase a regulation of the hepatic glycogen-targeted protein phosphatase 1 improves glucose tolerance in vivo. Cell. Signal. 21, 1123–1134. doi: 10.1016/j.cellsig.2009.03.001, 19275933

[ref7] Kelsall I. R. Voss M. Munro S. Cuthbertson D. J. Cohen P. T. (2011). R3F, a novel membrane-associated glycogen targeting subunit of protein phosphatase 1 regulates glycogen synthase in astrocytoma cells in response to glucose and extracellular signals. J. Neurochem. 118, 596–610. doi: 10.1111/j.1471-4159.2011.07345.x 21668450

[ref8] Liu Z. Xin B. Smith I. N. Sency V. Szekely J. Alkelai A. . (2023). Hemizygous variants in protein phosphatase 1 regulatory subunit 3F (PPP1R3F) are associated with a neurodevelopmental disorder characterized by developmental delay, intellectual disability and autistic features. Hum. Mol. Genet. 32, 2981–2995. doi: 10.1093/hmg/ddad124, 37531237 PMC10549786

[ref9] Piton A. Gauthier J. Hamdan F. F. Lafrenière R. G. Yang Y. Henrion E. . (2011). Systematic resequencing of X-chromosome synaptic genes in autism spectrum disorder and schizophrenia. Mol. Psychiatry 16, 867–880. doi: 10.1038/mp.2010.54, 20479760 PMC3289139

[ref10] Richards S. Aziz N. Bale S. Bick D. Das S. Gastier-Foster J. . (2015). Standards and guidelines for the interpretation of sequence variants: a joint consensus recommendation of the American College of Medical Genetics and Genomics and the Association for Molecular Pathology. Genet. Med. 17, 405–424. doi: 10.1038/gim.2015.30 PMC4544753 25741868

[ref11] Sobreira N. Schiettecatte F. Valle D. Hamosh A. (2015). GeneMatcher: a matching tool for connecting investigators with an interest in the same gene. Hum. Mutat. 36, 928–930. doi: 10.1002/humu.22844, 26220891 PMC4833888

[ref12] Verbinnen I. Ferreira M. Bollen M. (2017). Biogenesis and activity regulation of protein phosphatase 1. Biochem. Soc. Trans. 45, 89–99. doi: 10.1042/BST20160154, 28202662

